# Variation of *Goliathus orientalis* (Moser, 1909) Elytra Nanostructurations and Their Impact on Wettability

**DOI:** 10.3390/biomimetics3020006

**Published:** 2018-04-04

**Authors:** Guilhem Godeau, René-Paul Godeau, François Orange, Caroline R. Szczepanski, Frédéric Guittard, Thierry Darmanin

**Affiliations:** 1 NICE Lab, Université Côte d’Azur, Parc Valrose, 06100 Nice, France; rp.godeau@club-internet.fr (R.-P.G.); caroline.szczepanski@unice.fr (C.R.S.); guittard@unice.fr (F.G.); 2 Centre Commun de Microscopie Appliquée (CCMA), Université Nice Sophia Antipolis, 06100 Nice, France; francois.orange@unice.fr

**Keywords:** natural surface, beetle, wettability, petal effect, nanostructures, bioinspiration

## Abstract

Among the different species of flower beetles, there is one of particular notoriety: the Goliath beetle. This large insect can grow up to 11 cm long and is well-known for its distinctive black and white shield. In this paper, we focus on a particular *Goliathus* species: *G. orientalis* (Moser, 1909). We investigated the variations in properties of both the black and white parts of the upper face of *G. orientalis*; more precisely, the variation in surface properties with respect to the wettability of these two parts. This work reveals that the white parts of the shield have a higher hydrophobic character when compared to the black regions. While the black parts are slightly hydrophobic (*θ* = 91 ± 5°) and relatively smooth, the white parts are highly hydrophobic (*θ* = 130 ± 3°) with strong water adhesion (parahydrophobic); similar to the behavior observed for rose petals. Roughness and morphology analyses revealed significant differences between the two parts, and, hence, may explain the change in wettability. The white surfaces are covered with horizontally aligned nanohairs. Interestingly, vertically aligned microhairs are also present on the white surface. Furthermore, the surfaces of the microhairs are not smooth, they contain nanogrooves that are qualitatively similar to those observed in cactus spines. The nanogrooves may have an extremely important function regarding water harvesting, as they preferentially direct the migration of water droplets; this process could be mimicked in the future to capture and guide a large volume of water.

## 1. Introduction

Insect species are ubiquitous and have a significant influence across most of the world. To colonize and survive, insects need to adapt to a wide diversity of environments. Included among these varied locales are aqueous domains, where native species must have the ability to swim efficiently through or traverse on top of a body of water; insect species must also navigate terrestrial environments, where the capacity to climb and crawl over rocks and plants is necessary, as well as gaseous atmospheric locales where species must traverse by flying with minimal drag or inhibition. Throughout centuries of evolution, insects have managed to adapt perfectly to various environmental stresses specific to these different locales. The success of these adaptations has made insects a very relevant subject of investigation, as well as an infinite source of inspiration for scientists [1,2,3,4,5,6,7,8,9]. In particular, the control of surface wettability is very important for mitigating and dealing with external stressors for several insects. For example, swimming insects need to be perfectly hydrophilic to preserve their hydrodynamic properties and move through an aqueous environment as efficiently as possible [3]. Additionally, insects that traverse across air/water interfaces need superhydrophobic legs to achieve this feat. Similarly, in atmospheric environments, flying insects need to protect their wings from water, dust, and other pollutants so as not to be overloaded, and in order to preserve their ability to fly [7,8,9,10,11,12,13,14]. When thinking about this myriad of design challenges, the question is, how did these insects manage to control their wettability for these varied environments?

In response to this query, theoretical formulations have been proposed to explain the ability of a surface to repel water, many of which are well-known among the research community. One of the oldest among these theories is the Young–Dupré equation, which predicts wettability for smooth, homogeneous surfaces [15]. To predict wettability on rough surfaces, two models can be employed. The first is that proposed by Wenzel, which describes the case where a water droplet deposited on a rough surface totally imbibes all the surface roughness features [16]. The second, well-known model, used to describe wettability on rough surfaces is the Cassie–Baxter equation [17]. This formulation describes the case where a water droplet remains on top of the surface roughness, and does not imbibe nor flow between features, leaving pockets of vapor trapped between the fluid and the interface. The Wenzel and Cassie–Baxter regimes are considered extreme states, and it must be noted that the intermediate wetting states also exist. Some have been studied in nature, one example being rose petals, which are characterized by high hydrophobicity (large contact angle with water) and extremely strong water adhesion [18,19,20]. This particular intermediate state of wettability, observed on the rose petal, has been studied in detail and coined parahydrophobicity [21]. When discussing different interfacial wetting regimes, it is also prudent to mention surfaces with heterogeneous character, thus contrasting differences in hydrophobicity/hydrophilicity that exist in nature. Certain insects and plants have evolved to have superhydrophobic and superhydrophilic domains patterned on a surface in order to efficiently collect water from the surrounding environment, one example being the Namib Desert beetle [22]. Jiang et al. also reported that the cactus *Opuntia microdasys* from the Chihuahuan Desert can also collect water from fog, due to their spines and their three-dimensional surface morphology [23,24,25,26]. The spines contain microgrooves with a higher roughness near the tip than near the base, creating a wettability gradient along the length of these features, drawing water towards the cactus skin. The understanding gained from observation and study of these natural examples (rose petals, desert beetle, and cactus spines), among many others, may allowto answer the posed question of how different insect species manage to control surface wettability. For insects, in order to control their wettability, they must simultaneously take advantage of two parameters: their surface energy and their surface morphology. The surface energy is typically altered via variation of the natural surface composition (i.e., wax cement), or the epicuticular secretion of different species [27]. Concerning the second parameter, varied surface morphologies have been reported to have a significant influence over wettability. Some examples include butterfly wings which have nicely structured scales, as well as water striders which have legs decorated with nanohairs.

**Figure 1:**
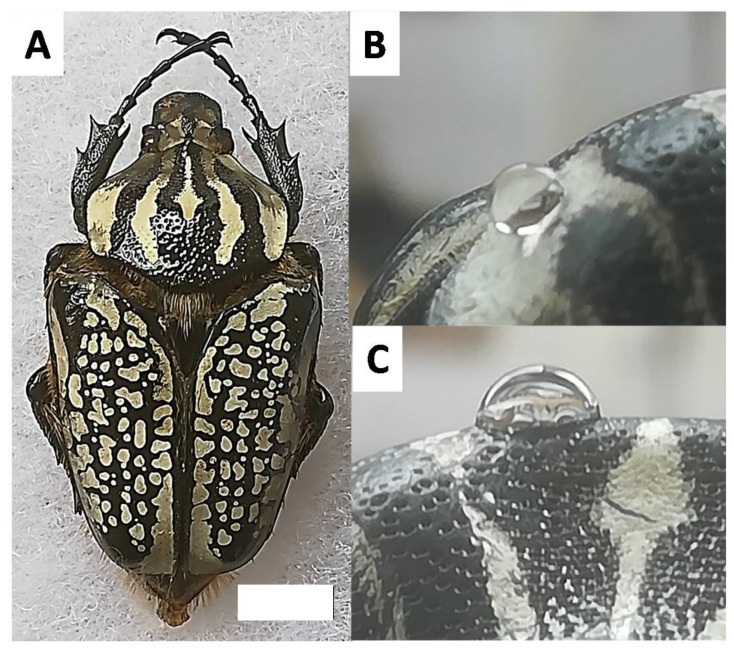
(**A**) Specimen of *Goliathus orientalis* (Moser, 1909); scale bar: 1 cm. (**B**) Water droplet deposited on the white part of the *G. orientalis* pronotum. (**C**) Water droplet deposited on the black part of the *G. orientalis* pronotum.

The control of surface wettability is not only a key element for insect survival and adaptation. A broad range of industrial applications that require a specific interfacial design exist. This includes anti-icing, antifogging, waterproofing, antibioadhesion, and water harvesting which are all deeply rooted in the need to understand and control wettability [13,28,29]. For this reason, investigations into unstudied insects such as the giant beetle, can be a source of original observations to provide new insights into types of surface chemistry or morphology that influence macroscopic interfacial behavior such as wettability. Giant beetles do not have typically sought-after properties, such as the ability to walk on aqueous surfaces, nor are their wings superhydrophobic. However, recent research based on these beetles has demonstrated an interesting connection between water and the insect cuticle surface. For example, color switching has been observed in the *Dynastes* genus depending on the air humidity [30]. With the awareness that closer investigation of giant beetles can inspire new materials, this work focuses on another remarkable example of giant beetles: the *Goliathus* genus. This genus was first described in 1801 by Lamarck and is very popular due to its unique appearance. The most well-known picture of the *Goliathus* is by far the image of a large black and white beetle (Figure 1A). Regardless of the external environmental conditions, the *Goliathus* specimen always presents this spectacular appearance with well-defined white markings. An interesting aspect of these white parts is that they remain clear and clean. This genus has been studied by many entomologists and is fully described [31,32]. However, up to date, the variation of the *Goliathus* wettability in relation to the black and white parts has not yet been described.

The main question of the study presented here is whether both the black and white features of the *G. orientalis* elytra have similar surface wettability, or rather the white part has particular or unique surface properties. Here, we present wettability and roughness measurements showing very significant wettability differences between the two parts (Figure 1B,C). The surface morphologies are also analyzed to provide insight into the function of this difference in wettability.

## 2. Materials and Methods

### 2.1. Dried Insect Specimens

All dried insects were purchased from Ets. Chaminade (Chaminade Entomologie, Sanary sur Mer, France). All animals used were fully-developed adults.

### 2.2. Sample Preparation

The dead insects were first immersed in water for 1 h to make the insects softer. The elytra were then detached and washed three times with ethanol. The samples were then allowed to dry overnight. The dried elytra were then cut before observation. Control observations were performed on native elytra.

### 2.3. Surface Characterization

All observations were performed on dried elytra. The apparent and dynamic contact angles were obtained with a DSA30 goniometer (Krüss, Hamburg, Germany). The apparent contact angles were measured using the sessile drop method and the dynamic contact angles were obtained using the tilted drop method. With the tilted drop method, a 2 μL water droplet was placed onto the elytron surface. The surface was then tilted until the droplet rolled off the surface. Surfaces where the water droplet did not roll off at inclination angles up to 90° were deemed parahydrophobic. The arithmetic mean roughness was measured via optical profilometry (Wyko NT 1100, Bruker, Billerica, MA, USA). The measurements were completed with a high mag phase shift interference (PSI), 50× objective, and a 0.5× field of view. All experiments were performed six times to acquire the standard deviation. Samples were coated with platinum and scanning electron microscopy (SEM) images were obtained with a JEOL 6700F scanning electron microscope.

## 3. Results

### 3.1. Materials

This work focused on the genus *Goliathus* (Lamarck, 1801). The members of this genus are quite popular and recognized worldwide due to their spectacular appearance. These insects are also known as the biggest flower beetles within the family Cetoniidae. While the genus *Goliathus* contains relatively few species, they are still of keen interest within the scientific community. All species of this genus have been characterized for their unique color, size, and layout of their markings. Most researchers today recognize five species [31,33,34,35]. Our work here focuses on the species *G. orientalis* (Moser, 1909). This species has been selected because of its large black and white patterns, which have yet to be investigated.

In order to isolate the surfaces suitable for observation, the elytra were removed. The elytra were then washed in ethanol and dried. Most of the surface secretions have been described as hardly soluble in ethanol [27]. However, to verify this claim, native elytra were also observed as control samples and produced the same results (data not shown). Therefore, it is assumed that the washing process does not significantly alter the surface properties.

### 3.2. Wettability of the Goliathus Elytra

**Figure 2:**
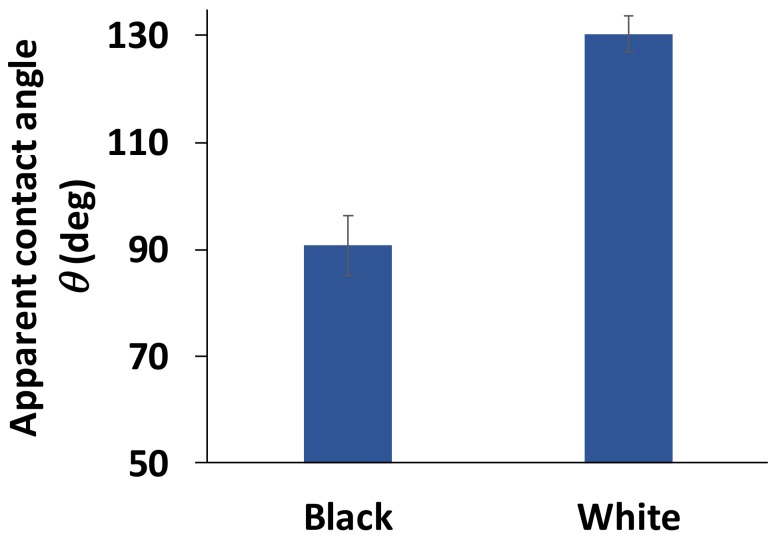
Wettability measurements on the black and white parts of the *G. orientalis* elytra. The graph represents mean ± standard deviation.

The first part of this work investigates the variation in *G. orientalis* elytra wettability on the black or white features of the elytra. The measurements were performed using water as a probe liquid, showing a very significant difference in wettability between these two features (Figure 2).

**Figure 3:**
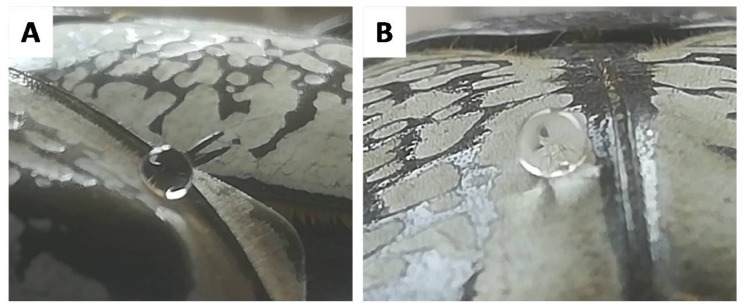
Water droplet deposited on the white part of a *G. orientalis* elytron. (**A**) Side view and (**B**) back view.

The apparent contact angle of water droplets indicates a very slight hydrophobicity of the black surfaces with *θ* = 91 ± 5°, while the wettability of the white part can be described as highly hydrophobic with *θ* = 130 ± 3°. Even though the quantitative measurement of the hydrophobicity of the white surface is already high, it is important to note that this value is actually underestimated, and the actual degree of hydrophobicity of the white surface is likely to be much higher (Figure 3).

**Figure 4:**
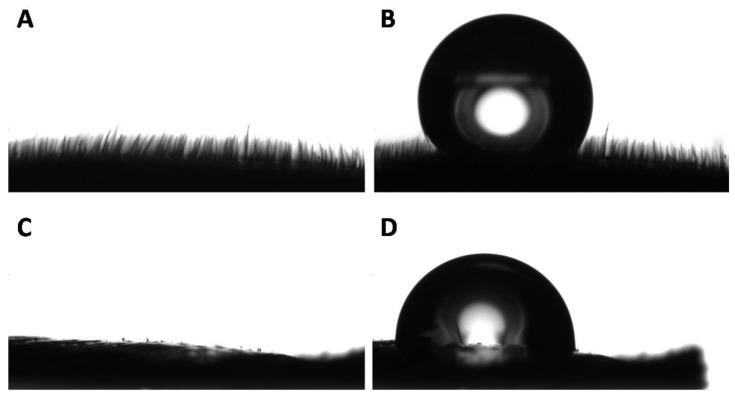
Representative side view of the white and black surfaces. (**A**) Profile of the white part of a *G. orientalis* elytron and (**B**) water droplet deposited on its surface. (**C**) Profile of the black part of a *G. orientalis* elytron and (**D**) water droplet deposited on its surface.

To further validate this claim, the side view of the white surface was observed with the goniometer camera and it revealed the presence of microscopic hairs that covered the entirety of the surface (Figure 4A). 

**Figure 5:**
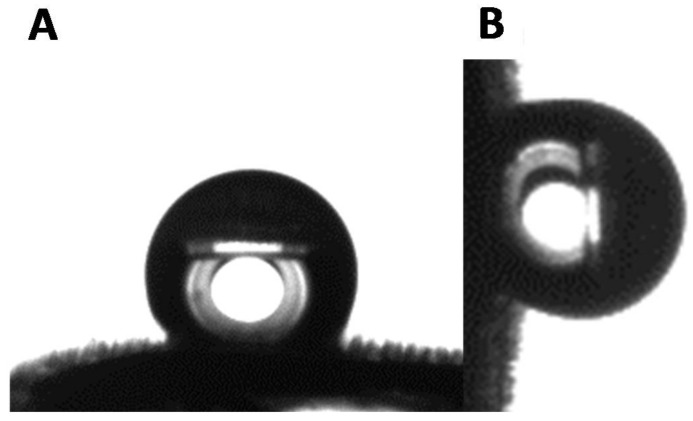
Example of parahydrophobic features observed on the white part of a *G. orientalis* elytron. Water droplet on (**A**) horizontal and (**B**) tilted surface.

The presence of microhairs on the white part made the observation and discernment of the triple point complicated precluding the accurate analysis of the contact angle (Figure 4B). Interestingly, the side observation of the black part of the elytron did not reveal hair-like microstructures (Figure 4C), and the apparent contact angle of the black part was measured with more accuracy (Figure 4D). To further understand these observations, dynamic measurements were performed on both black and white parts. Not surprisingly, considering the high contact area provided by the microhairs on the white part, and the relatively low degree of hydrophobicity on the black features, both surfaces showed high adhesion of water and can be described as parahydrophobic (Figure 5). A water droplet would remain stuck to the surface even if the surface was tilted by 90°. This precise combination of static and dynamic wetting behavior is also known as the petal effect in the literature [18,19,20,21]. This property is often exploited in nature when water harvesting and collection is necessary for survival [22,23,24,25,26,27,28,29,30,31,32,33,34,35,36].

### 3.3. Surface Roughness of the Goliathus Elytra

**Figure 6:**
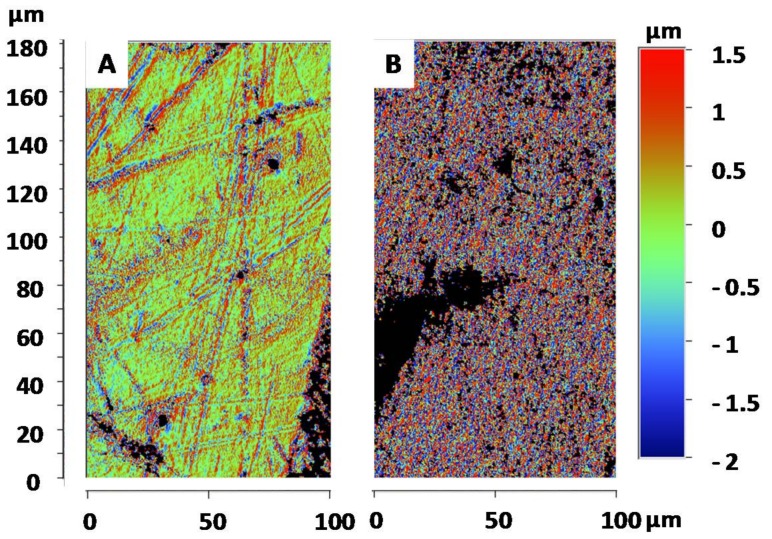
Representative profilometer images observed for the (**A**) white and (**B**) black parts of the *G. orientalis* elytra.

To confirm these observations regarding the differences between the black and white surfaces, roughness measurements were performed. The measurements revealed a significant difference between the black (Figure 6A) and the white (Figure 6B) surfaces.

**Figure 7:**
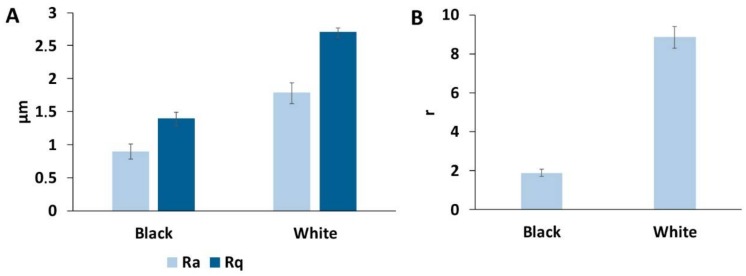
Roughness measurements of the *G. orientalis* elytra surface. (**A**) Arithmetic mean roughness (Ra) and quadratic mean roughness (Rq) measurement. (**B**) Calculated roughness (r) parameter.

With these analyses, the arithmetic mean roughness (Ra) and quadratic mean roughness (Rq) were measured for both black and white surfaces (Figure 7A). For the black surface, Ra and Rq were 0.9 and 1.4 µm, respectively; and for the white surface, Ra and Rq were 1.8 and 2.7 µm, respectively. These results are consistent with the previous observation and show a significant difference between the black and white surfaces. Additionally, the roughness parameter employed in the Wenzel equation (cos(*θ^w^*) = r cos(*θ^y^*)) was also determined (Figure 7B).

This calculation reveals once again the large difference in surface roughness, with r = 1.9 and r = 8.8 for black and white surfaces, respectively.

### 3.4. Surface Morphologies of the Goliathus Elytra

**Figure 8:**
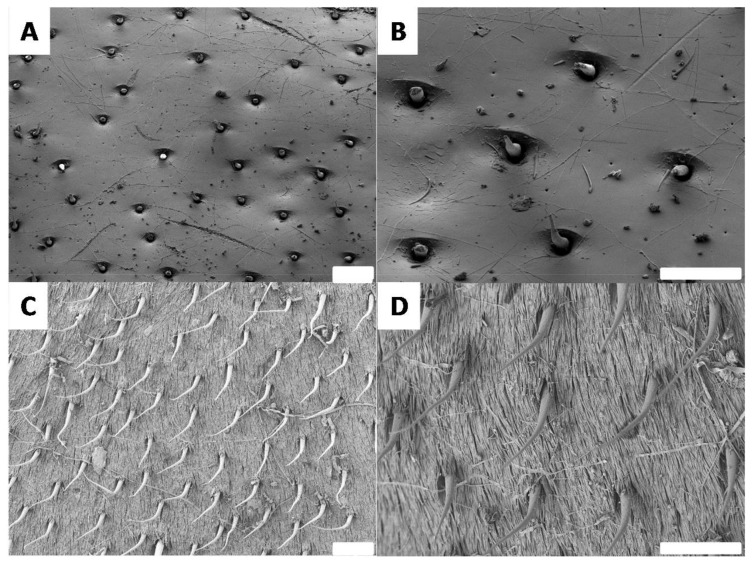
Scanning electron microscopy images showing the morphology of the *G. orientalis* (**A**,**B**) black and (**C**,**D**) white surfaces. Scale bar: 100 µm.

In order to completely understand the differences in surface properties, it is necessary to investigate the surface morphology. Morphological observations were performed by SEM. First, the black and white surfaces were observed using low magnification (Figure 8).

**Figure 9:**
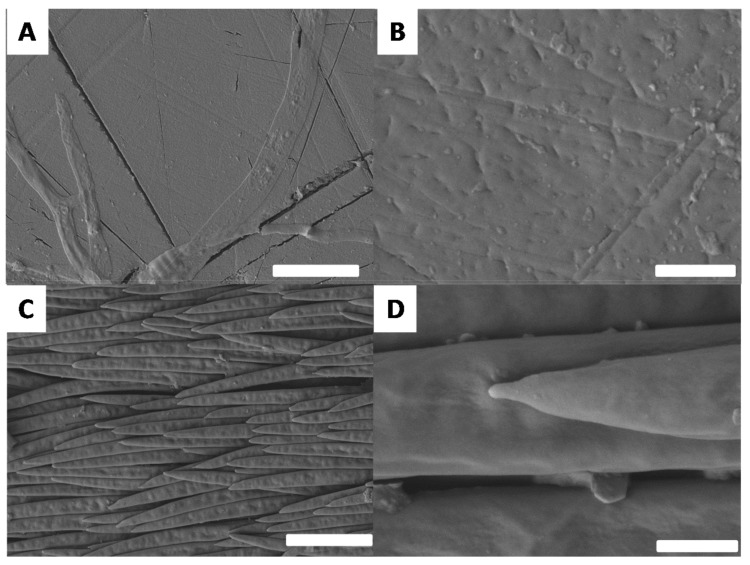
Scanning electron microscopy images showing the morphology of the *G. orientalis* (**A**,**B**) black and (**C**,**D**) white surfaces. Scale bars: 10 µm (**A**,**C**); 1 µm (**B**,**D**).

These observations were consistent with all previous measurements. The black surface appears to have very few and relatively short structures at a very low surface density (Figure 8A,B). In contrast, the white surface is homogeneously covered by microhairs (Figure 8C). On closer inspection, it is also possible to notice that the surface covered by microhairs is itself structured (Figure 8D). To further investigate these morphologies, both the black and white surfaces were analyzed by SEM at a higher magnification (Figure 9).

As expected, the analysis of higher magnification SEM images revealed the presence of a smooth surface in the black part (Figure 9A,B). In the white part, the surface of the elytron (shown in Figure 8C) is covered with horizontally aligned nanohairs (Figure 9C,D). Compared to the vertical hairs, the horizontal hairs are thinner and much flatter. Such a morphology is quite consistent with the structures described for other insects with white features [37]. In the case of *G. orientalis*, the double structuration is very consistent with the high degree of hydrophobicity observed for the white part. Furthermore, the smooth surface of the black part supports the lower hydrophobicity observed for this surface. 

**Figure 10:**
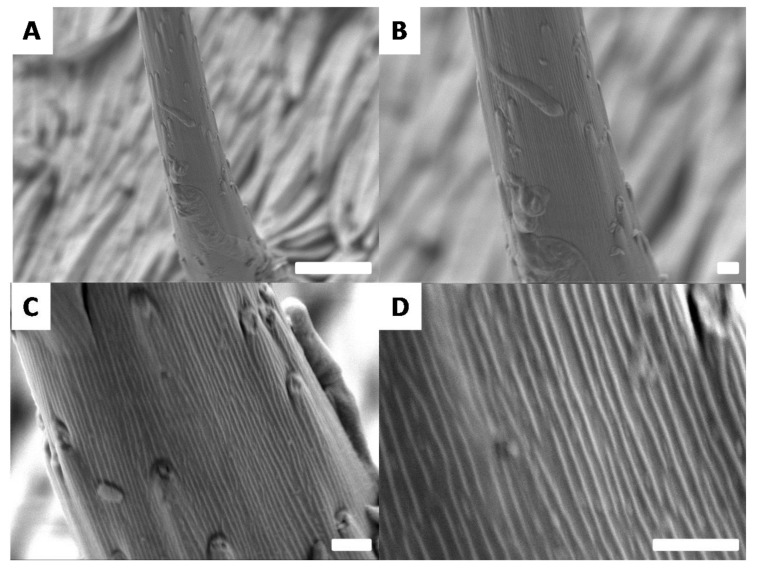
Scanning electron microscopy images showing the morphology of a *G. orientalis* microhair. Scale bars: 10 µm (**A**); 1 µm (**B**–**D**).

To complete the study of the white surface, additional SEM analysis were performed on the vertically aligned microhairs (Figure 10).

The vertical microhairs were observed at different magnifications (Figure 10A–D). Scanning electron microscopy images revealed a very interesting structure. The microhairs appear to be covered by horizontally aligned nanogrooves. (Figure 10C,D). These structures have been regularly observed in nature, particularly on species with the ability to harvest water which have developed structures, such as the cactus spines, for the directional movement of water droplets [23,24,25,26]. 

Combined with the parahydrophobic behavior of the *G. orientalis* surface, our data suggest that the white part may play an important role in directing water for this animal. The microhairs are conical in shape, and the spacing between the nanogrooves is different at the bottom and the top of the microhairs, which is similar to the hierarchical structure observed on the cactus spines. As a consequence, a gradient in both the surface roughness and hydrophobicity may allow water droplets to migrate from the top of the microhairs to their base in one direction and coalesce into the less hydrophobic surface of the insect containing the horizontally aligned nanohairs, which in turn may act to slowly spread the droplets on the surface, similar to the behavior reported for cacti [23,24,25,26]. This observation is also consistent with the large white stripe observed on the *G. orientalis* pronotum, which may play a role in directing water to the head of the animal.

## 4. Conclusions

The present work showed very significant differences in the surface properties between the black and the white parts of the *G. orientalis*. While the black parts were slightly hydrophobic (*θ* = 92°), the white parts were highly hydrophobic (*θ* = 131°) with strong water adhesion (parahydrophobic character), similar to what is observed on rose petals. The analysis of the surface roughness and morphology shed light on the hypothesis that the difference in surface wettability observed may be related to the different structures found on the elytra surface. Indeed, the black part is relatively smooth whereas the white surface is covered with horizontally aligned nanohairs. Furthermore, vertically aligned microhairs are also present on the white surface of the elytra. The surface of these microhairs appears not to be smooth, but instead it is covered with nanogrooves similar to the hierarchical structures observed on cactus spines. The hierarchical nanogroove structure on the surface of the *G. orientalis* elytra may inspire the development of novel water harvesting systems and applications in the future. Hence, with this design, water droplets could be captured and guided with ease for directional water transport.
